# An Updated Systematic and Comprehensive Review of Cytoreductive Prostatectomy for Metastatic Prostate Cancer

**DOI:** 10.3390/curroncol30020170

**Published:** 2023-02-10

**Authors:** Takafumi Yanagisawa, Pawel Rajwa, Tatsushi Kawada, Kensuke Bekku, Ekaterina Laukhtina, Markus von Deimling, Muhammad Majdoub, Marcin Chlosta, Pierre I. Karakiewicz, Axel Heidenreich, Takahiro Kimura, Shahrokh F. Shariat

**Affiliations:** 1 Department of Urology, Comprehensive Cancer Center, Medical University of Vienna, Wahringer Gurtel 18-20, 1090 Vienna, Austria; 2 Department of Urology, The Jikei University School of Medicine, Tokyo 105-8461, Japan; 3 Department of Urology, Medical University of Silesia, 41-800 Zabrze, Poland; 4 Department of Urology, Okayama University Graduate School of Medicine, Dentistry and Pharmaceutical Sciences, Okayama 700-8530, Japan; 5 Institute for Urology and Reproductive Health, Sechenov University, 119435 Moscow, Russia; 6 Department of Urology, University Medical Center Hamburg-Eppendorf, 20251 Hamburg, Germany; 7 Department of Urology, Hillel Yaffe Medical Center, 169, Hadera 38100, Israel; 8 Clinic of Urology and Urological Oncology, Jagiellonian University, 30-688 Krakow, Poland; 9 Cancer Prognostics and Health Outcomes Unit, Division of Urology, University of Montreal Health Center, Montreal, QC H2X 0A9, Canada; 10 Department of Urology, Faculty of Medicine and University Hospital of Cologne, 50937 Cologne, Germany; 11 Division of Urology, Department of Special Surgery, The University of Jordan, Amman 19328, Jordan; 12 Department of Urology, University of Texas Southwestern Medical Center, Dallas, TX 75390, USA; 13 Department of Urology, Second Faculty of Medicine, Charles University, 15006 Prague, Czech Republic; 14 Department of Urology, Weill Cornell Medical College, New York, NY 10021, USA; 15 Karl Landsteiner Institute of Urology and Andrology, 1090 Vienna, Austria

**Keywords:** metastatic prostate cancer, prostatectomy, cytoreductive prostatectomy, local therapy

## Abstract

(1) Background: Local therapy is highly promising in a multimodal approach strategy for patients with low-volume metastatic prostate cancer (mPCa). We aimed to systematically assess and summarize the safety, oncologic, and functional outcomes of cytoreductive prostatectomy (cRP) in mPCa. (2) Methods: Three databases were queried in September 2022 for publications that analyzed mPCa patients treated with cytoreductive prostatectomy without restrictions. The outcomes of interest were progression-free survival (PFS), cancer-specific survival (CSS), overall survival (OS), perioperative complication rates, and functional outcomes following cRP. (3) Results: Overall, 26 studies were included in this systematic review. Among eight population-based studies, cRP was associated with a reduced risk of CSS and OS compared with no local therapy (NLT) after adjusting for the effects of possible confounders. Furthermore, one population-based study showed that cRP reduced the risk of CSS even when compared with radiotherapy (RT) of the prostate after adjusting for the effects of possible confounders. In addition, one randomized controlled trial (RCT) demonstrated that local therapy (comprising 85% of cRP) significantly improved the prostate-specific antigen (PSA)-PFS and OS. Overall, cRP had acceptable perioperative complication rates and functional outcomes. (4) Conclusions: Mounting evidence suggests that cRP offers promising oncological and functional outcomes and technical feasibility and that it is associated with limited complications. Well-designed RCTs that limit selection bias in patients treated with cRP are warranted.

## 1. Introduction

The management of metastatic hormone-sensitive prostate cancer (mHSPC) has transformed during the past decade owing to the emergence of combination systemic therapies, such as androgen receptor signaling inhibitor and/or docetaxel plus androgen deprivation therapy (ADT) [1,2,3,4,5,6]. Further, mHSPC is a heterogeneous disease entity with varied prognoses. Tumor burden stratified as low- vs. high-volume disease (defined as the presence of visceral metastases, or four or more bone metastases, of which at least one must be located outside the vertebral column or pelvic bone) on the basis of the definition of the CHAARTED trial has been shown to stratify mHSPC into different risk categories [4,6,7,8,9,10,11]. For mHSPC patients with low-volume disease, local destructive therapy for primary prostate cancer and metastasis-directed therapy have gained widespread use [12]. For example, the STAMPEDE trial showed an OS benefit by delivering radiation therapy (RT) to the prostate in mHSPC patients treated with a standard of care for low-volume disease [13]. Since then, there has been increasing interest in local therapy (LT) as a part of the treatment strategy for mHSPC to ensure durability in efficacy and quality of life (i.e., local progression prevention). In addition to RT, cytoreductive prostatectomy (cRP) has been used in this setting. A previous meta-analysis based on population-based studies demonstrated the OS benefit of cRP even in mHSPC patients, including both low- and high-volume diseases [14]. Despite this, there are several limitations in the methodology of the published literature, primarily owing to heterogeneity in the included studies and patient selection [14,15,16]. One such factor is the even-increasing heterogeneity in the low-volume mHSPC group. On the basis of the increasing implementation of prostate-specific membrane antigen (PSMA)–positron emission tomography (PET) scans in clinical practice, an increasing number of oligometastatic patients with mHSPC are being identified [17]. Therefore, we conducted this systematic and comprehensive review to update and assess the safety, oncologic, and functional outcomes in mHSPC patients who underwent cRP.

## 2. Materials and Methods

The protocol has been registered in the International Prospective Register of Systematic Reviews database (PROSPERO: CRD42022368246).

### 2.1. Search Strategy

This systematic review was carried out according to the guidelines of the Preferred Reporting Items for Meta-Analyses of Observational Studies in Epidemiology Statement (Supplementary Figure S1) [18]. A literature search on the PubMed, Web of Science, and Scopus databases was performed in September 2022 to identify studies investigating the perioperative, oncologic, or functional outcomes of cRP for mPCa. The detailed search strategy was as follows: (metastatic) AND (prostate cancer) AND (prostatectomy) OR (cytoreductive). The primary outcomes of interest were overall survival (OS) and cancer-specific survival (CSS), and the secondary outcomes of interest were progression-free survival (PFS), perioperative outcomes, and urinary and erectile functional outcomes. The initial screening based on the titles and abstracts aimed to identify eligible studies and was performed by two investigators. Potentially relevant studies were subjected to a full-text review. Disagreements were resolved by consensus with the coauthors.

### 2.2. Inclusion and Exclusion Criteria

Studies were included if they investigated metastatic PCa (mPCa) patients (patients), who underwent cRP (interventions) compared with those treated with RT or without LT (comparisons), to assess the differential oncologic, perioperative, and functional outcomes (outcome) in randomized controlled studies (RCTs) and in nonrandomized, observational, population-based, or cohort studies (Study design). We excluded studies that compared the differential outcomes of LT vs. non-LT (NLT), not separately reporting the outcomes of cRP or RT unless more than 80% of patients treated with LT were cRP. Studies lacking original patient data, reviews, letters, editorial comments, replies from authors, case reports, and articles not written in English were excluded. References of all papers included were scanned for additional studies of interest.

### 2.3. Data Extraction

Two authors independently extracted the following data: the first author’s name, publication year, country, inclusion criteria, number of patients, follow-up duration, age, performance status or comorbidity, clinical stage, biopsy Gleason score (GS), pretreatment of prostate-specific antigen (PSA), metastatic site, surgical approach of cRP, lymph node dissection (LND) and number of removed lymph nodes (LNs), estimated blood loss, operation time, catheterization periods, length of hospital stay, all and severe (≧Clavien-Dindo classification Ⅲ) postoperative complication rates, positive surgical margin (PSM), LN involvement, pathologic stage, GS in the resected specimen, continence rates, erectile function, patient-reported quality of life (QOL), OS, CSS, PFS, time to castration-resistant prostate cancer (CRPC), and CRPC-free survival. Subsequently, the hazard ratios (HRs) and 95% confidence intervals (CIs) from Cox regression models for OS and CSS were retrieved. All discrepancies were resolved by consensus with the coauthors.

### 2.4. Risk-of-Bias Assessment

The study quality and risk of bias were assessed according to the Risk of Bias in Nonrandomized Studies of Interventions (ROBINS-I) tool and the risk-of-bias (RoB version2), referring to the Cochrane Handbook for Systematic Reviews of Interventions [18]. Each bias domain and the overall risk of bias were judged as ‘low’, ‘moderate’, ‘serious’ or ‘critical’. The presence of possible confounders was determined by consensus and a literature review. The ROBINS-I and risk-of-bias assessment of each study were independently conducted by two authors (Supplementary Figure S2 and Table S1).

## 3. Results

### 3.1. Study Selection and Characteristics

**Figure 1:**
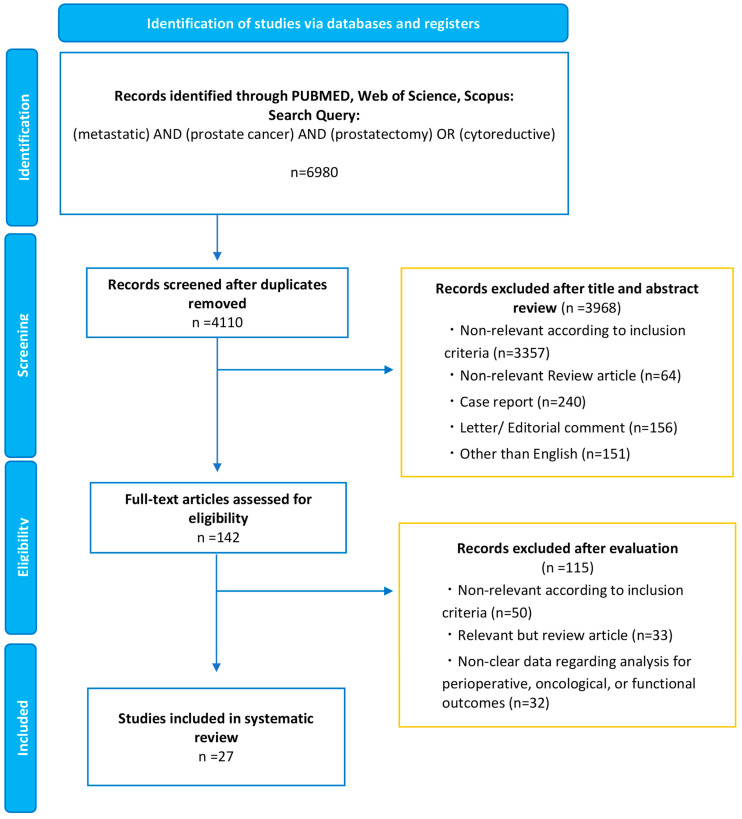
The Preferred Reporting Items for Systematic Reviews and Meta-analyses (PRISMA) flow chart, detailing the article-selection process.

Our initial search identified 6980 records. After removing duplicates, 4110 records remained for screening titles and abstracts (Figure 1). After screening, a full-text review of 142 articles was performed. According to our inclusion criteria, we finally identified 27 studies eligible for systematic review [19,20,21,22,23,24,25,26,27,28,29,30,31,32,33,34,35,36,37,38,39,40,41,42,43,44,45]. The demographics of each included study are shown in Table 1 and Table 2. Of the 27 studies, 9 were population-based designs, 11 were comparative (including case-control cohorts and RCTs), and seven included only cRP patients. This section may be divided by subheadings. It should provide a concise and precise description of the experimental results, their interpretation, and the experimental conclusions that can be drawn.

### 3.2. Oncologic Outcomes

#### 3.2.1. Population-Based Studies

We identified seven studies by using the Surveillance, Epidemiology, and End Results (SEER) database and one study each by using the Munich Cancer Registry database and the National Cancer Database (NCDB). SEER and NCDB reflect the real-world survival data of patients diagnosed with mPCa in the US. However, variables unavailable from SEER, such as patient performance status, comorbidity, and metastatic burden (i.e., including both high- and low-volume disease), undoubtedly limited the granularity and generalizability of the analyses and precluded controlling for the often-existent selection bias [20]. Patient demographics of included population-based studies are summarized in Supplementary Table S2.

##### cRP vs. NLT

In 2014, Culp et al. for the first time showed the OS and CSS benefit of LT for mPCa among 8185 mPCa patients (NLT: *n* = 7811, cRP: *n* = 245, brachytherapy [BT]: *n* = 129) by using the SEER database from 2004 to 2010 [20]. The authors reported that the 5-year OS and CSS were significantly higher in patients undergoing cRP (67.4% and 75.8%, respectively) or BT (52.6% and 61.3%, respectively) compared with those without LT (22.5% and 48.7%, respectively) [20]. After adjusting for the effects of confounders, such as TNM stage and PSA, using multivariable competing risks regression analysis, statistical significance remained (HR for cRP: 0.38 [95% CI: 0.27–0.53], HR for BT: 0.68 [95% CI: 0.49–0.93]) [20]. Thereafter, Antwi et al. and Satkunasivam et al. performed the additional analyses using propensity scores (PS) in 2014 and 2015 [19,26]. Antwi et al. showed that PS-adjusted HR for OS in patients who underwent cRP was 0.22 (95% CI: 0.17–0.28) compared with those without LT [19]. Satkunasivam et al. assessed the same oncologic outcomes in patients 66 years or older (*n* = 4069) [26]. Owing to the low number of patients who underwent cRP (*n* = 47), PS-adjusted HR for OS did not reach statistical significance (HR: 0.55 [95% CI: 0.30–1.02]) [26]. In 2017, Parikh et al. published results from the NCDB comprising 6051 patients (NLT: *n* = 5224, cRP: *n* = 622, radiotherapy [RT]: *n* = 205) by adjusting for the effects of confounders such as age, TN stage, and the Charlson comorbidity index (CCI) [25]. The authors showed that the adjusted HR by using the Cox proportional hazard model for OS in patients who underwent cRP was 0.51 (95% CI: 0.45–0.59) and confirmed the OS benefit of cRP even after PS adjustment (HR: 0.27 [95% CI: 0.22–0.33]) [25].

Since 2020, two studies using the SEER database have been published. Jin et al. updated the study period (2010–2014) from the study conducted by Culp et al., comprising 5849 patients (NLT: *n* = 5628, cRP: *n* = 159, BT: *n* = 62) [24]. The authors corroborated previous findings suggesting an OS and CSS benefit by using the Cox proportional hazard models (HR: 0.60 [95% CI: 0.42–0.87], HR: 0.56 [95% CI: 0.37–0.86], respectively) [24]. In addition, a subgroup analysis revealed that patients with bone metastasis or distant LN metastasis were significantly more likely to benefit from definitive local therapy [24]. Despite the limitation of selection bias derived from a population-based study, the detailed analyses adjusting for the effects of confounders revealed an OS and CSS benefit for cRP over NLT.

##### cRP vs. RT

Jin et al. compared oncologic outcomes by using the SEER database (2004–2015) comprising 19,612 patients (NLT: *n* = 18,857, cRP: *n* = 435, RT: *n* = 320) [23]. The authors confirmed the OS and CSS benefit of LT over NLT even after adjusting for the effects of unmeasured confounders (HR for OS: 0.57 [95% CI 0.50–0.65], HR for CSS: 0.59 [95% CI 0.51–0.68], respectively.) [23]. Furthermore, the authors showed that cRP was associated with significantly better OS and CSS compared with RT after adjusting for the effects of race, age, marital status, TNM stage, GS, and PSA as well as performance status [23]. However, after adjusting for the effects of unmeasured confounders, this statistical significance diminished (HR for OS: 0.63 [95% CI 0.26–1.54] and HR for CSS: 0.47 [95% CI 0.16–1.35], respectively) [23]. Guo et al. created 1:1 PS-matched cohorts (cRP: *n* = 148, RT: *n* = 148) based on data from the SEER database (2004–2016) [22]. The authors failed to show the superiority of cRP over RT in terms of OS (HR: 0.73 [95% CI: 0.48–11]) and CSS (HR: 0.77 [95% CI: 0.46–1.30]) [22]. A recently published study using the SEER database (2004–2016) conducted by Stolzenbach et al. comprised 954 patients who underwent cRP and 3326 patients who underwent RT [27]. Despite short follow-up periods (median follow-up was within 2 years), they showed that cRP is associated with significantly better CSS compared with RT after adjusting for age, initial PSA, biopsy GS, and clinical TNM stages using PS and the competing risk regression (HR: 0.82 [95% CI: 0.71–0.94]) [27]. However, the results from the SEER database differ according to the recruitment periods, statistical methods, and follow-up duration, leaving the potential benefits of cRP over RT controversial.

#### 3.2.2. Case-Control Studies

Patient demographics and oncologic outcomes of included studies are shown in Table 2 and Supplementary Table S3.

##### cRP vs. NLT

Six case-control studies assessing the differential oncologic outcomes were identified. In 2015, Heidenreich et al. assessed the differential oncologic outcomes of cRP (*n* = 23) vs. NLT (*n* = 38) in patients with oligometastatic mPCa (less than three bone metastases) with comparable patient demographics except for baseline PSA [33]. The authors reported significantly better PFS, time to CRPC, and CSS in patients who underwent cRP compared with those who did not undergo LT [33]. In 2017, Poelart et al. reported the preliminary results of the LoMP trial with extremely better oncologic outcomes in terms of 100% of 2-year CSS and OS in patients who underwent cRP (*n* = 17) compared with patients without LT (*n* = 29). On the other hand, Moschini et al. and Steuber et al. showed no differences in CSS or OS between patients who underwent cRP and those who did not [39,42]. In addition, updated results from the LoMP trial comprising 40 patients in each arm (NLT vs. cRP) showed no differences in CRPC-free survival on multivariable analysis [29]. However, most recently, Mistretta et al. demonstrated that NLT was associated with higher rates of progression to mCRPC (HR: 0.40; CI 0.19–0.84 adjusted for the effects of the site of metastasis, HR:0.39; CI 0.19–0.84 adjusted for the effect of PSA) while adjusting only for one confounder [38]. Taken together, there is conflicting evidence on the oncologic benefit of cRP from case-control studies. Notably, these studies included only 17 to 43 patients in the cRP group; therefore, these studies suffered from low statistical power. We initially attempted to perform a meta-analysis to integrate them; however, these studies also suffered different inclusion criteria and unmatched comparators. This suggests the need for well-controlled future trials and for international collaborative multicenter studies with more patients.

##### cRP vs. RT

Lumen et al. reported comparable oncological outcomes between cRP and RT from the LoMP trial [36]. Comparing cRP (*n* = 48) vs. RT (*n* = 26), the 2-year CSS were 93% vs. 100%, and the 2-year OS were 93% vs. 100%, respectively [36]. Knipper et al. compared the oncologic outcomes of cRP in mHSPC patients with low-volume disease and the results from the STAMPEDE arm H [13,35]. The authors showed comparable 3-year OS and CSS rates for cRP and RT (OS: 91% vs. 81%, CSS: 92% vs. 86%, respectively) [35]. To date, high-quality evidence regarding the differential oncological outcomes between cRP and RT is lacking; nonetheless, the oncological effectiveness of cRP with PLND may be comparable to pelvic RT.

#### 3.2.3. RCT

Up to now, only one RCT, conducted by Dai et al., has been published [31]. The authors conducted an open-label phase-2 RCT to compare the oncologic outcomes between LT (*n* = 100) and NLT (*n* = 100). The LT group comprised 85 (85%) patients who underwent cRP and 11 (11%) patients who underwent RT, whereas 17 patients (17%) eventually received LT in the NLT group. This study showed significantly better OS (HR: 0.44 [95% CI: 0.24–0.81]), radiographic PFS (HR: 0.43 [95% CI: 0.27–0.70]), and PSA-PFS (HR: 0.44 [95% CI: 0.29–0.67]) in patients who underwent LT compared with those who did not during 48 months of median follow-up [31].

### 3.3. Perioperative Outcomes

#### 3.3.1. Complications

Assessing perioperative outcomes on the basis of different surgical approaches is imperative. Robot-assisted radical prostatectomy (RARP) has recently replaced open and laparoscopic approaches as the standard technique [46,47]. Traditionally, cRP has been performed by using an open approach [33,39,45], while recently, RARP has become the standard procedure even for cRP (Table 3) [29,38,40,41,43].

In total, after combining patients from all studies, there were 155 overall complications reported in 473 patients (33%) and 47 severe complications (CTCAE grade≥ 3) reported in 448 patients (10%) who underwent cRP. Only one rectal injury was reported in 347 patients (0.29%). However, most eligible studies did not report outcome data on cRP for either open or robot-assisted approaches, making meaningful comparisons challenging. The largest multicenter cohort reported by Heidenreich et al. (open: *n* = 104, RARP: *n* = 5) showed that the rates of overall and severe complications were 34% and 9.7%, respectively [32].

For open cRP alone, the overall and severe complication rates ranged from 29% to 54% and from 6.5% to 21%, respectively [33,35,39,45]. In comparison, Sooriakumaran et al. reported a 12.5% overall complication rate in patients who underwent cytoreductive RARP (cRARP) [41]; furthermore, Takagi et al., assessing the feasibility of cRARP in 12 patients, reported excellent perioperative outcomes without any complications [43]. Taken together, cRARP seems safer than open cRP, in agreement with the previously demonstrated safety of RARP for localized PCa [48].

#### 3.3.2. Pathologic Outcomes

Of the studies included, 15 provided data on the rates of PSM, ranging from 8.3% to 82% [28,29,30,31,32,33,34,37,39,40,41,42,43,44,45]. Most studies performed concomitant PLND during cRP; 15 studies provided data on the rates of LN involvement, ranging from 31% to 91% [28,29,30,31,32,33,34,37,39,40,41,42,44,45]. The wide range of PSM rates suggests the importance of optimal patient selection and the need for adjuvant RT in some patients. Extended PLND should be performed during cRP given the high likelihood of LN involvement.

### 3.4. Functional Outcomes

#### 3.4.1. Urinary Function

##### Obstructive Voiding Dysfunction in NLT Patients

Obstructive voiding dysfunction and relevant lower urinary tract symptoms (LUTSs) due to the local progression of PCa are critical clinical issues in the late stages of mPCa [49]. Evaluating the intervention rates for obstructive urinary dysfunction in NLT patients, a retrospective case-control study by Heidenreich et al. showed that 11 of 38 (29%) patients required surgical or percutaneous intervention [33]. The LoMP trial conducted by Poelaert et al. revealed that 11 of 29 (38%) NLT patients required intervention [29]. In addition, Steuber et al. reported that 14 of 40 (35%) NLT patients experienced severe local complications [42]. Notably, Lumen et al. demonstrated that cRP was associated with higher local event-free rates than NLT on multivariable analysis (HR: 0.36 [95% CI: 0.14–0.94]) [36]. Preventing obstructive voiding dysfunction seems to be an essential rationale for undergoing cRP for mPCa patients in the earlier disease stages before disease progression.

##### Incontinence after cRP

The timing and tools for assessing urinary incontinence after cRP varied across studies (Table 4). Continence rates, defined as pad 0–1/day 1 year after cRP, ranged from 74% to 88% [28,29,35]. Of note, 0 pad achievement rates at 1-year follow-up after cRP ranged from 53% to 92% [28,29,31,32,35]. For example, Knipper et al. showed that 53% who underwent open cRP (*n* = 78) did not use any pad/day [35]. Furthermore, another large multicenter study conducted by Heidenreich et al. comprising 113 patients (92% of patients underwent open cRP) found a 68% 0 pad rate at 1-year follow-up [32]. In the recent RCT conducted by Dai et al., excellent continence rates, of 92%, at 1 year and 95% at 2 years after cRP were reported, although only 20% of patients underwent cRARP [31]. There are no robust data regarding urinary function following cRARP.

Interestingly, Chaloupka et al. compared the functional outcomes between cRP (open cRP: *n* = 69, cRARP: *n* = 13) and RP for localized PCa (open RP: *n* = 116, RARP: *n* = 216) [30]. This study revealed comparable continence recovery rates (66% vs. 72%, *p* = 0.4) as well as International Consultation on Incontinence Questionnaire scores (ICIQ-SF; 6.4 ± 5.7 vs. 6.4 ± 5.2 [mean ± SD], *p* = 1) at 25 months after surgery [30].

#### 3.4.2. Erectile Function

Two studies assessed erectile function before and after cRP using the International Index of Erectile Function (IIEF)-5 score [30,41]. The TroMbone trial revealed comparable IIEF-5 scores between the cRP and NLT groups (Median [IQR]: 5.0 [5.0–6.0] vs. 5.0 [5.0–12.0]) [41]. On the contrary, a study conducted by Chaloupka et al. comparing the functional outcomes between cRP and RP for localized PCa (Open cRP: *n* = 116, cRARP: *n* = 216) showed that the IIEF-5 score was significantly lower in the patients who underwent cRP compared with those who underwent RP for localized PCa (mean ± SD: 1.3 ± 4.2 vs. 3.5 ± 6.2, *p* < 0.001) [30]. The low rates of nerve sparing cRP (cRP: 17% vs. RP for localized PCa: 55%) indeed affect these outcomes [30].

#### 3.4.3. Quality of Life

The TroMbone trial also assessed the patient-reported QOL using the EuroQoL Five Dimensions Five Levels (EQ-5D-5L) questionnaires at baseline and 3 months postrandomization [41]. This study showed a comparative EQ-5D-5L descriptive score at 3 months after randomization between the cRP and NLT groups (median [IQR]: both 1.0 [0.8–1.0]) [41]. Chaloupka et al. compared the general health-related QOL (HRQOL) by global health status (GHS) by using the European Organization for Research and Treatment of Cancer (EORTC) quality-of-life questionnaire (QLQ)-C30 between cRP and RP for the localized PCa group. This study demonstrated no difference in the general HRQOL rates between the two groups at the end of follow-up (44% vs. 56%, *p* = 0.8) [30]. Interestingly, GHS significantly worsened in localized PCa patients compared with the baseline (–5, *p* = 0.001), whereas GHS did not significantly change in patients who underwent cRP (+3.2, *p* = 0.4) [30]. Taken together, cRP seems not to reduce the patient-reported QOL compared with patients with NLT.

## 4. Conclusions

Population-based studies showed an oncologic benefit to cRP compared with NLT or RT for mPCa, after careful analyses that adjusted for the effects of possible confounders. Nevertheless, these studies suffered from selection bias and lacked relevant data often used for clinical decision-making, such as comorbidity and metastatic burden. Small case-control studies, including only patients with oligometastatic disease, failed to report a clear survival benefit for cRP. Recently, only one RCT, including 85% of cRP patients in the LT group, demonstrated an oncologic benefit of LT in terms of PSA-PFS as well as OS. Perioperative and functional outcomes following cRP seem to be comparable to those of NLT or RP for localized PCa. Taken together, cRP offers promising oncological outcomes, technical feasibility, and acceptable functional outcomes. However, well-designed, adequately powered RCTs with long-term follow-ups are needed to allow a robust and fair comparison of cRP with NLT and RT. Until then, cRP should be considered experimental and assessed only in clinical trials.

## Figures and Tables

**Table 1:** Demographics and oncologic outcomes of population-based study.

Author	Year	Comparisons	No. of Patients	Recruitment Year	Inclusion Criteria	Confounders for Matching	Follow-Up	Cancer-Specific Mortality		Overall Mortality	
Culp et al. [20] (SEER)	2014	NLT	7811	2004–2010	·Stage IV (M1a-c) PCa (adenocarcinoma) at diagnosis identified using SEER and divided on the basis of definitive treatment of the RP or BT or NLT	ND	Median: 16 mo. (IQR: 7–31)	5-yearCSS: 48.7%		5-year OS: 22.5% (95% CI: 21.1–23.9)	
		cRP	245					5-yearCSS: 75.8%		5-year OS: 67.4% (95% CI: 58.7–74.7)	
		BT	129					5-yearCSS: 61.3%		5-year OS: 52.6% (95% CI: 39.8–63.9)	
Gratzke et al. [21] (Munich Cancer Registry)	2014	NLT	1075	1998–2010	ND	ND	ND	ND		5-year OS: 21%	
		cRP	74							5-year OS: 55%	
		RT	389							ND	
Antwi et al. [19] (SEER)	2014	NLT	7516	2004–2010	·Stage IV (M1a-c) PCa (adenocarcinoma) at diagnosis identified using SEER and divided on the basis of definitive treatment of the RP or BT or NLT	Age, race, marital status, tumor grade, PSA level, and cancer registry	ND	ND	Reference	ND	Reference
		cRP	222						Adjusted HR: 0.28 (95% CI: 0.20–0.39)		Adjusted HR: 0.27 (95% CI: 0.20–0.38)
		BT	120						Adjusted HR: 0.46 (95% CI: 0.33–0.64)		Adjusted HR: 0.43 (95% CI: 0.31–0.59)
Satkunasivam et al. [26] (SEER)	2015	NLT	3827	2004–2009	·Stage IV (M1a-c) PCa (adenocarcinoma) at diagnosis identified using SEER and divided on the basis of definitive treatment of the RP or IMRT or CRT or NLT ·Included only patients > age 65 years	Age at diagnosis, diagnosis year, race, marital status, pretreatment PSA (categorical variable), clinical tumor stage and grade, CCI, ADT, and bone radiation within 6 months of diagnosis	Median: 20 mo. (IQR: 10–36)	3-year CSS: 46%	Reference	3-year OS: 34%	Reference
		cRP	47					3-year CSS: 79%	Adjusted HR: 0.48 (95% CI: 0.27–0.85)	3-year OS: 73%	Adjusted HR: 0.43 (95% CI: 0.26–0.70)
		IMRT	88					3-year CSS: 82%	Adjusted HR: 0.38 (95% CI: 0.24–0.61)	3-year OS: 72%	Adjusted HR: 0.45 (95% CI: 0.31–0.65)
		CRT	107					3-year CSS: 49%	Adjusted HR: 0.85 (95% CI: 0.64–1.14)	3-year OS: 37%	ND
Parikh et al. [25] (NCDB)	2017	NLT	5224	2004–2013	·Stage IV (M1a-c) PCa (adenocarcinoma) at diagnosis identified using NCDB and divided on the basis of definitive treatment of the RP or IMRT or CRT or NLT	Race, age, CCI score, T-stage, N-stage, insurance status, income quartile, facility type, and use of ADT	Median: 22 mo.	ND	ND	5-year OS: 17.1%	Reference
		cRP	622							5-year OS: 51.4%	Adjusted HR: 0.51 (95% CI: 0.45–0.59)
		CRT	153							5-year OS: 26.8%	Adjusted HR: 1.04 (95% CI: 0.86–1.27)
		IMRT	52								Adjusted HR: 0.47 (95% CI: 0.31–0.72)
Jin S et al. [24] (SEER)	2020	NLT	5628	2010–2014	·Stage IV (M1a-c) PCa (adenocarcinoma) at diagnosis identified using SEER and divided on the basis of definitive treatment of the RP or BT or NLT	ND	ND	ND	Reference	ND	Reference
		cRP	159						Adjusted HR: 0.56 (95% CI: 0.37–0.86)		Adjusted HR: 0.60 (95% CI: 0.42–0.87)
		BT	62						Adjusted HR: 0.71 (95% CI: 0.43–1.18)		Adjusted HR: 0.72 (95% CI: 0.46–1.14)
Jin K et al. [23] (SEER)	2020	NLT	18,857	2004–2015	·Stage IV (M1a-c) PCa (adenocarcinoma) at diagnosis identified using SEER and divided on the basis of definitive treatment of the RP or RT or NLT ·Patients who received EBRT with unknown region were excluded	marital status, race, age, clinical TNM stages, GS, and PSA level	ND	Reference	NA	Reference	NA
		cRP	435					Adjusted HR (Cox regression): 0.39 (95% CI: 0.34–0.45) Adjusted HR (PSM): 0.50 (95% CI: 0.41–0.60) Adjusted HR (Covariate adjustment PS): 0.57 (95% CI:0.49–0.66)	Adjusted HR (Cox regression): 0.61 (95% CI: 0.42–0.91) Adjusted HR (PSM): 0.49 (95% CI: 0.32–0.73)	Adjusted HR (Cox regression): 0.39 (95% CI: 0.35–0.44) Adjusted HR (PSM): 0.51 (95% CI: 0.44–0.60) Adjusted HR (Covariate adjustment PS): 0.57 (95% CI: 0.50–0.65)	Adjusted HR (Cox regression): 0.60 (95% CI: 0.43–0.83) Adjusted HR (PSM): 0.45 (95% CI: 0.32–0.65)
		RT	320						Reference		Reference
Guo et al. [22] (SEER)	2021	cRP	481 (148) *	2004–2016	·Stage IV (M1a-c) PCa (adenocarcinoma) at diagnosis identified using SEER and divided on the basis of definitive treatment of the RP or RT ·Patients with incomplete clinicopathological data, such as T-stage, PSA value, and GS were excluded	Age, year of diagnosis, PSA level, clinical tumor stage, biopsy GS, and the M stage	Median (IQR): 37 mo. (14.0–83.5)	10-year CSS: 73.8%	Adjusted HR (PSM): 0.77 (95% CI: 0.46–1.30) Adjusted HR (SMRW): 0.83 (95% CI: 0.52–1.32)	10-year OS: 60.8%	Adjusted HR (PSM): 0.73 (95% CI: 0.48–1.11) Adjusted HR (SMRW): 0.75 (95% CI: 0.52–1.09)
		RT	203 (148) *				Median (IQR): 56.5 mo. (18.0–110.0)	10-year CSS: 66.7%	Reference	10-year OS: 45.6%	Reference
Stolzenbach et al. [27] (SEER)	2021	cRP	954	2004–2016	·Stage IV (M1a-b) PCa (adenocarcinoma) at diagnosis identified using SEER and divided on the basis of definitive treatment of the RP or RT ·On the basis of the composition of the STAMPEDE trial and on the basis of the definition of low-volume mPCa, M1c patients were excluded	Age at diagnosis, initial PSA, biopsy GGG, and clinical T, N and M1 stages	Median (range): 23 mo. (11–46)	PSM cohort 5-year CSM: 47% (*p* = 0.003)	Adjusted HR (PSM and CRR): 0.79 (95% CI: 0.68–0.90)	ND	ND
		RT	3326				Median (range): 21 mo. (10–42)	PSM cohort 5-year CSM: 53% (*p* = 0.003)	Reference		

No.; number, Pts.: patients, NCDB; National Cancer Database, SEER; surveillance, epidemiology and end results, LT; local therapy, NLT: no local therapy, RT; radiotherapy, EBRT; external beam radiotherapy, BT; brachytherapy, IMRT; intensity-modulated radiotherapy, CRT; conformal radiation therapy, PS; propensity score, PSM; propensity-score matching, CRR; competing risks regression, SMRW; standardized mortality ratio weighting, cRP; cytoreductive radical prostatectomy, IQR; interquartile range, NA; not applicable, ND; No data, PSA; prostate-specific antigen, GS; Gleason score, GGG: Gleason grade group, CSS; cancer-specific survival, CSM; cancer-specific mortality, ACM; all-cause mortality, OS; overall survival, HR; hazard ratio, CI; confidence interval, PCa; prostate cancer, CCI; Charlson comorbidity index. * Described as number of PSM cohorts.

**Table 2:** Study demographics and oncologic outcomes of cohort studies.

Author	Year	Comparisons	No. of pts.	Recruitment Year	Country (Institution)	Inclusion Criteria	Treatment after cRP	Follow-Up Duration	Progression-Free	Time to CRPC/CRPC-Free Survival	Cancer-Specific Survival	Overall Survival
Comparative studies between cRP and no cRP												
Heidenreich et al. [33]	2015	cRP	23	ND	Germany	Patients with biopsy-proven PCa, minimal bone metastases (3 or fewer hot spots on bone scan), absence of visceral or extensive LN metastases and PSA decrease to less than 1.0 ng/mL after neoadjuvant ADT	No treatment: 9 (39) ADT only: 5 (21): ABI: 5 (21) DOC: 2 (21)	Median (range): 34.5 mo. (7–75)	Median (range): 38.6 mo. (12–52)	Median (range): 40 mo. (9–65)	Median: 47 mo. (range: 9–71) 95.6%	91.3%
		No cRP	38			Patients with mPCa treated with ADT without LT served as the control group		Median (range): 37.0 mo. (28–96)	Median (range): 26.5 mo. (12–48)	Median (range): 29 mo. (16–54)	Median: 40.5 mo. (range: 19–75) 84.2%	78.9%
Poelaert et al. [40] (LoMP trial)	2017	cRP	17	2014-	Multicenter	RP was performed in asymptomatic patients with a resectable tumor and who were fit to undergo surgery (group A, *n* = 17)	No treatment: 4 (24) ADT: 13 (76)	Mean ± SD: 13 ± 8 mo.	ND	No patients develop CRPC	2-yr: 100%	2-yr: 100%
		No cRP	29			Only SOC was administered to patients with mPCa ineligible or unwilling to undergo cRP (group B, *n* = 29)		Mean ± SD: 16 ± 10 mo.		Median (range): 14 mo. (2–26)	2-yr: 61%	2-yr: 55%
Moschini et al. [39]	2017	cRP	31	2007–2014	USA (Mayo)	31 (66%) underwent cRP with or without adjuvant therapies and 16 (34%) underwent ADT only	M1a: minimum 6 mo. ADT M1b: minimum 6 mo. ADT + MDT	Median: 38.8 mo.	ND	ND	1-yr: 100% 3-yr: 91.3% 5-yr: 61.9%	ND
		No cRP	16				NA				1-yr: 93.8% 3-yr: 76.9% 5-yr: 46.2%	
Steuber et al. [42]	2017	cRP	43	2000–2011	Germany (Martini-Klinik Prostate Cancer Center)	Patients with low-volume bone metastases (1–3 lesions) undergoing cRP	All patients received ADT or CAB	Median: 32.7 mo.	ND	No significant differences in CRPC-free survival: *p* = 0.92	ND	No significant differences in overall survival: *p* = 0.92
		No cRP	40			Patients receiving best systemic therapy		Median: 82.2 mo.				
Buelens et al. [29] (LoMP trial)	2022	cRP	40	2014–2018	Multicenter	Asymptomatic patients: cRP was offered to all fit patients with resectable tumors, resulting in 40 patients; standard of care was administered to 40 patients who were ineligible or unwilling to undergo surgery	All patients received ADT ± DOC/ABI	Median (IQR): 38 (32–50) mo.	ND	Median CRPC-free survival: 53 mo. (95% CI: 14–92) 3-yr: 59% (95% CI: 43–74)	ND	ND
		No cRP	40					Median (IQR): 31 (15–46) mo.		Median CRPC-free survival: 21 mo. (95% CI: 15–27) 3-yr: 40% (95% CI: 25–55)		
Mistretta et al. [38]	2022	cRP	40	2010–2018	Italy	Patients affected by cM1a-b oligometastatic PCa (defined as <5 metastatic lesions at diagnosis involving M1a and/or bone (M1b), with locally resectable cT1-T3 tumors)	Adjuvant ADT at least 12 mo.	Median: 55 mo.	radiological progression: 83.1%	mCRPC rate: 24.0%	CSM: 5.9%	ND
		No cRP	34				NA	Median: 50 mo.	radiological progression: 62.5%	mCRPC rate: 62.5%	CSM: 37.1%	
RCT (Phase1/2) for cRP vs. No cRP												
Sooriakumaran et al. [41] (TRoMbone)	2022	cRP	25	ND	UK (multicenter)	Patients diagnosed with oligometastatic PCa (defined as one to three skeletal lesions on bone imaging, no visceral metastases); locally resectable tumor (clinical/radiological stage T1–T3; ECOG-PS 01); and suitable for RP within 3 months of starting SOC All patients received SOC systemic therapy of ADT + DOC	All patients received ADT ± DOC	ND	ND			
		No cRP	25						ND			
Comparative studies between cRP and RT												
Knipper et al. [35]	2020	cRP	78	2008–2018	Germany (Martini-Klinik Prostate Cancer Center)	Patients with newly diagnosed mPCa with low-volume (<4 bone metastases) and no visceral metastases according to STAMPEDE definition), confirmed on bone scan and CT/MRI, who underwent RP with PLND	All patients received ADT	Median (IQR): 36 (15–48) mo.	3-yr metastatic PFS: 63%	ND	3-yr CSS: 92%	3-yr OS: 91%
		STAMPEDE arm H (low volume with RT)	410			NA	NA	ND	3-yr metastatic PFS: 67%		3-yr CSS: 86%	3-yr OS: 81%
RCT (Phase2) assessing LT (including 85% of cRP) vs. NLT												
Dai et al. [31]	2022	LT	100 (85) *	2015–2019	China	Patients with newly diagnosed oligometastasis PCa defined as five or fewer bone or extrapelvic LN metastases and no visceral metastases	All patients received ADT (94% received CAB)	Median (IQR): 48 (43–50) mo.	Median rPFS: not reached 3-yr rPFS: 79% HR: 0.43, 95% CI: 0.27–0.70	ND	ND	3-yr OS: 88% HR: 0.44, 95% CI: 0.24–0.81
		NLT	100						Median rPFS: 40 mo. 3-yr rPFS: 56%			3-yr OS: 70%
Comparative studies between cRP, RT, and NLT												
Lumen et al. [36] (LoMP trial)	2021	cRP	48	2014-	Multicenter	RP was performed in asymptomatic patients with a resectable tumor and who were fit to undergo surgery; only SOC was administered to patients with metastatic prostate cancer ineligible or unwilling to undergo cRP For this study, patients with high-volume disease were excluded, leaving only patients with low-volume disease for evaluation	All patients received SOC (ADT ± ARSI or DOC)	Median (IQR): 42 (24–57) mo.	ND	ND	2-yr CSS: 93% (vs. NLT: HR 0.36, 95% CI:0.14–0.94)	2-yr OS: 93% (vs. NLT: HR 0.28, 95% CI:0.11–0.71)
		RT	26					Median (IQR): 26 (14–51) mo.			2-yr CSS: 100% (vs. NLT: HR 0.33, 95% CI: 0.09–1.20)	2-yr OS: 100% (vs. NLT: HR 0.26, 95% CI: 0.07–0.91)
		NLT	35					Median (IQR): 24 (12–44) mo.			2-yr CSS: 75%	2-yr OS: 69%
Comparative studies between cRP and RP for localized PCa												
Chaloupka et al. [30]	2021	cRP	79	2012–2020	Germany (Ludwig-Maximilians University)	cRP was performed in patients with oligometastasis, defined as <5 bone lesions in the preoperative staging; biopsy-proven PCa, history of RP at one tertiary center and completed follow-up; patients with preoperative ADT and pre-RP RT of the prostate were excluded from further analysis Of 1268 pts., matched cohort of 411 patients were retained after PSM	ND	ND	ND	ND	5-yr CSS: 61%	5-yr OS: 38%
		RP for localized PCa	332								5-yr CSS: 81%	5-yr OS: 57%
Single arm or only including cRP cohort												
Gandaglia et al. [45]	2017	cRP	11	2006–2011	Italy	Patients with oligometastatic PCa	Adjuvant ADT: 10 (91)	Median (IQR): 63 mo. (48–77)	7-yr cPFS: 45%	ND	7-yr CSS: 82%	ND
Heidenreich et al. [32]	2018	cRP	113	ND	Multicenter	Biopsy-proven mPCa who fulfilled the following selection criteria: (1) completely resectable PCa; (2) osseous metastases; (3) absence of gross retroperitoneal LN metastases; (4) absence of bulky pelvic LN metastases >3 cm; (5) no or minimal visceral metastases; (6) ECOG-PS of 0–1; and (7) written informed consent	Neoadjuvant ADT:80 (71), adjuvant ADT: 91 (87)	Median (range): 45.7 mo. (13–96)	Median(range): 72.3 mo. (8–96) 65 pts. remain clinical progression-free at 5yr	ND	ND	3-yr OS: 89.3% 5-yr OS: 80.5%
Xue et al. [44]	2020	cRP + MDT	26	2012–2016	Multicenter (China)	(1) Biopsy-confirmed diagnosis of prostate adenocarcinoma; (2) M1b disease with the presence of 1–5 visible bone metastases (by Tc-99m MDP BS, CT, or MRI); (3) not received RT and chemotherapy in hormone-sensitive phase; (4) adequate organ function; (5) ECOG performance status 0.1; (6) pretreatment total testosterone > 200 ng/dL; and (7) written informed consent	All patients received ADT	Median (range): 43.1 mo. (15–61)	ND	3-yr CRPC-free survival: 75.9% RP + MDT had better CRPC-free survival (HR: 0.41, 95% CI: 0.18–0.95)	3-yr CSS: 91.4% No significant differences between two groups (HR: 0.59, 95% CI: 0.12–2.95)	ND
		cRP only	32					Median (range): 47.6 mo. (18–65)				
Mandel et al. [37] (ProMPT trial)	2021	cRP (assessing CTC as prognostic value)	33	2014–2015	Germany (Martini-Klinik Prostate Cancer Center)	(1) Newly diagnosed PCa, with 1–3 bone metastases (positive BS and confirmed by CT or MRI; no PETCT was used) at the time of diagnosis; (2) asymptomatic patient; (3) absence of visceral metastases; (4) locally resectable tumor (<cT3); (5) PSA at diagnosis <150 mg/dl; and (6) no prior radiation of bone metastases; in addition to cRP, the best systemic therapy (only ADT) was recommended to all patients	All patients were recommended ADT	Median: 39.4 mo.	ND	3-yr CRPC-free survival: 65.6%	ND	3-yr OS: 87.9%
Babst et al. [28]	2021	cRP (assessing the impact of upfront DOC-based doublet therapy)	38	2015–2018	Germany (two centers)	Patients with mHSPC underwent cRP after primary chemohormonal therapy (DOC +ADT)	All patients received ADT continuously	Median (range): 22.6 mo. (5.7–48.6)	ND	Median time to CRPC: 35.9 mo.	ND	ND
Kim et al. [34]	2022	cRP	32	ND	Multicenter (USA, South Korea, Japan)	The major inclusion criterion was biopsy-proven N1M0 or NxM1a/b PCa	All patients received ADT	Median (IQR): 46 (32–53) mo.	ND	ND	ND	5-yr OS: 67% (All patients) 69% (M1 patients)
Takagi et al. [43]	2022	cRP (assessing the feasibility of RARP)	12	2017–2021	Japan (two centers)	Patients with mPCa who had undergone neoadjuvant therapy followed by RARP	Adjuvant ADT: 5 (25)	ND	BCR-free survival: 1-yr: 83.3%/2-yr: 66.7% MFS: 1-yr: 90%/2-yr: 90%	ND	ND	ND

No.: number, Pts.: patients, LT: local therapy, NLT: no local therapy, RT: radiotherapy, EBRT: external beam radiotherapy, BT: brachytherapy, MDT: metastasis-directed therapy, PS: propensity score, PSM: propensity-score matching, ECOG-PS: Eastern Cooperative Oncology Group Performance Status, cRP: cytoreductive radical prostatectomy, CRPC: castration-resistant prostate cancer, SOC: standard of care, RARP: robot-assisted radical prostatectomy, IQR: interquartile range, SD: standard deviation, ADT: androgen deprivation therapy, CAB: combined androgen blockade, ARSI: androgen receptor signaling inhibitor, DOC: docetaxel, NA: not applicable, ND: no data, BCR: biochemical recurrence, CSS: cancer-specific survival, CSM: cancer-specific mortality, OS: overall survival, HR: hazard ratio, CI: confidence interval, PCa: prostate cancer, mPCa: metastatic PCa, CTC: circulating tumor cell, BS: bone scan. * 85 patients underwent cRP.

**Table 3:** Perioperative outcomes following cRP.

Author	Year	Comparisons	No. of Pts.	Approach of CRP, *n* (%)	Nerve Sparing	LND, *n* (%)	LN Removed, *n*	LN Involvement, *n* (%)	Estimated Blood Loss, mL	Operation Time, Min	Catheterization, Days	LOS, Days	Postoperative Complication (All), *n* (%)	Postoperative Complication (CD > 3), *n*(%)	Rectal Injury, *n* (%)	PSM, *n* (%)
Heidenreich et al. [33]	2015	cRP	23	Open RP	ND	All ePLND	ND	13 (57)	Mean (range): 335 (250–600)	Mean (range): 127 (115–145)	Mean (range): 5.6 days (5–12)	Mean (range): 7.8 (6–13)	9 (39)	3 (13)	ND	4 (17)
Poelaert et al. [40] (LoMP trial)	2017	cRP	17	Open: 1 RARP: 16	None	All ePLND	Median (range): 20 (9–47)	12 (71)	Median (range): 250 (100–900)	Median (range): 215 (150–290)	ND	ND	7 (41)	0	0	14 (82)
Moschini et al. [39]	2017	cRP	31	Open RP	ND	All ePLND	Median (IQR): 19 (13–31)	20 (64)	Median (IQR): 400 (250–500)	ND	ND	Median (IQR): 3 (3–5)	30 days: 9 (29) 90 days: 4 (13)	30 days: 2( 6.5) 90 days: 2 (6.5)	0	8 (26)
Steuber et al. [42]	2017	cRP	43	ND	None: 74% Unilateral: 16% Bilateral: 9.3%	ND	Median (IQR): 21 (12–27)	67%	ND	ND	ND	ND	ND	ND	ND	67%
Buelens et al. [29] (LoMP trial)	2022	cRP	40	Open: 2 RARP: 38	None	All ePLND	Median (IQR): 17 (11–21)	31 (78)	Median (IQR): 250 (150–325)	Median (IQR): 205 (165–220)	ND	ND	90 days 20 (50)	2 (5)	0	32 (80)
Sooriakumaran et al. [41] (TRoMbone)	2022	cRP	25	RARP	ND	All ePLND	11 (7–14)	11 (46)	ND	Median (IQR): 185 (165–217)	14 (10–14)	Median (IQR): 1 (1–2)	3 (12.5)	ND	ND	10 (42)
Knipper et al. [35]	2020	cRP	78	Open RP	ND	ND	ND	ND	ND	ND	ND	ND	34 (44)	16 (21)	ND	ND
Dai et al. [31]	2022	cRP	85	Open RP: 68 (80) RARP: 17 (20)	ND	ND	ND	26 (31)	ND	ND	ND	ND	24 (28)	7 (8.2)	1 (1.2)	36 (42)
Chaloupka et al. [30]	2021	cRP	79	Open: 69 (87) RARP:10 (13)	13 (17)	ND	Median (IQR): 10 (6–13)	41 (52)	ND	ND	ND	ND	ND	ND	ND	59 (75)
		RP for localized PCa	332	Open: 116 (35) RARP: 216 (65)	183 (55)		Median (IQR): 11 (6–18)	112 (34)								165 (50)
Gandaglia et al. [41]	2017	cRP	11	Open	ND	All ePLND	Median (IQR): 27 (23–42)	10 (91)	Median (IQR): 750 (600–850)	Median (IQR): 170 (160–380)	ND	Median (IQR): 13 (7–19)	6 (54)	2 (18)	0	8 (73)
Heidenreich et al. [32]	2018	cRP	113	Open: 104 (92) RARP: 9 (8)	ND	None: 1.8% Limited: 8.8% Extended: 89.4%	Median (range): 15.3 (0–57)	70 (62)	ND	Median (range): 145 (95–380)	ND	Median (range): 6.5 (3–21)	38 (34)	11 (9.7)	0	42 (37)
Xue et al. [44]	2020	cRP + MDT	26	Open/LRP	ND	All	ND	10 (39)	ND	ND	ND	ND	ND	ND	ND	3 (12)
		cRP only	32					10 (32)								4 (13)
Mandel et al. [37] (ProMPT trial)	2021	cRP	33	Open: 28 (85) RARP: 5 (15)	ND	ND	ND	24 (73)	ND	ND	ND	ND	ND	ND	ND	24 (73)
Babst et al. [28]	2021	cRP	38	Open: 35 (92) RARP: 3 (7.9)	ND	All	Median (IQR): 18.5 (12–24)	34 (89)	ND	Median (IQR): 196 (157–233)	ND	Median (IQR): 9 (6–10)	within 30 days 5 (13)	within 30 days 4 (11)	0	21 (55)
Kim et al. [34]	2022	cRP	32	ND	ND	ND	ND	20 (62)	Median (IQR): 200 (100–400)	Median (IQR): 225 (198–312)	ND	ND	ND	6%	ND	20 (66)
Takagi et al. [43]	2022	cRP	12	RARP	None	None	ND	ND	Median (IQR): 23 (7–45)	Median (IQR): 85 (70–112) *	ND	ND	0	0	0	1 (8.3)

No.: number, Pts.: patients, MDT: metastasis-directed therapy, RP: radical prostatectomy, PCa: prostate cancer, cRP: cytoreductive radical prostatectomy, RARP: robot-assisted radical prostatectomy, IQR: interquartile range, SD: standard deviation, ADT: androgen deprivation therapy, NA: not applicable, ND: no data, LOS: length of stay, LN: lymph node, LND: lymph node dissection, ePLND: extended pelvic lymph node dissection, PSM: positive surgical margin, focal therapy. * Described as console time.

**Table 4:** Functional outcomes following cRP.

Author	Year	Comparisons	No. of Pts.	Approach of cRP, *n* (%)	Nerve Sparing	Pre-RP Urinary Function, *n* (%)	Continence during Follow-Up, *n* (%)	Pre-cRP Erectile Function, *n* (%)	Erectile Function at Last Follow-Up, *n* (%)
Heidenreich et al. [33]	2015	cRP	23	Open RP	ND	ND	at last follow-up 0 pads/day: 13 (57) 0–1 pads/day: 21 (91) 2–4 pads/day: 2 (8.7)	ND	ND
Poelaert et al. [40] (LoMP trial)	2017	cRP	17	Open: 1 RARP: 16	None	ND	at 3 mo. Continent and no local symptoms: 12 (71)	ND	ND
		No cRP	29	NA			at 3 mo. Continent and no local symptoms: 13 (45)		
Moschini et al. [39]	2017	cRP	31	Open RP	ND	ND	at 90 days 0 pads/day: 24 (77) 1–2 pads/day: 2 (6.5) >2 pads/day: 5 (16)	ND	ND
Buelens et al. [29] (LoMP trial)	2022	cRP	40	Open: 2 RARP: 38	None	ND	Continent (0–1 pads/day) at 1 yr: 31 (79) at last follow-up: 35 (88)	ND	ND
Sooriakumaran et al. [41] (TRoMbone)	2022	cRP	25	RARP	ND	Incontinence: 0	Incontinence at 1 mo.: 9 (37.5%) Incontinence at 3 mo.: 6 (25%) Incontinence at 6 mo.: 4 (17%)	IIEF-5 score (median, IQR): 13.0 (5.5–21.0)	IIEF-5 score at 3 mo. (median, IQR): 5.0 (5.0–6.0)
		No cRP	25	NA		Incontinence: 0	ND	IIEF-5 score (median, IQR): 18.5 (10.0–21.0)	IIEF-5 score at 3 mo. (median, IQR): 5.0 (5.0–12.0)
Knipper et al. [35]	2020	cRP	78	Open RP	ND	ND	at 1 yr 0 pads/day: 20 (53) 0–1 pads/day: 28 (74) 2 pads/day: 2 (5)	ND	ND
Dai et al. [31]	2022	cRP	85	Open RP: 68 (80) RARP: 17 (20)	ND	ND	at 1 yr 0 pads/day: 78 (92) at 2 yrs 0 pads/day: 81 (95)	ND	ND
Chaloupka et al. [30]	2021	cRP	79	Open: 69 (87) RARP:10 (13)	13 (17)	ICIQ-SF score (mean ± SD): 2.3 ± 4.6	at 25 mo. ICIQ-SF score (mean ± SD): 6.4 ± 5.7 Daily pad usage (mean ± SD): 1.6 ± 2.5 Continence recovery: 66%	IIEF-5 score (mean ± SD): 8.5 ± 10.2 IIEF-5 score > 18: 26.8%	IIEF-5 score (mean ± SD): 1.3 ± 4.2 IIEF-5 score > 18: 2.0%
		RP	332	Open: 116 (35) RARP: 216 (65)	183 (55)	ICIQ-SF score (mean ± SD): 1.1 ± 2.6	at 25 mo. ICIQ-SF score (mean ± SD): 6.4 ± 5.2 Daily pad usage (mean ± SD): 1.2 ± 1.7 Continence recovery: 72%	IIEF-5 score (mean ± SD): 11.3 ± 9.9 IIEF-5 score > 18: 37.2%	IIEF-5 score (mean ± SD): 3.5 ± 6.2 IIEF-5 score > 18: 6.8%
Gandaglia et al. [45]	2017	cRP	11	Open	ND	ND	at 90 days 0 pads/day: 3 (27)	ND	ND
Heidenreich et al. [32]	2018	cRP	113	Open: 104 (92) RARP: 9 (8)	ND	ND	at 12 mo. 0 pads/day: 68% 1–2 pads/day: 18% >2 pads/day: 14%	ND	ND
Babst et al. [28]	2021	cRP	38	Open: 35 (92) RARP: 3 (7.9)	ND	ND	0–1 pads/day at 1 mo.: 87% at 6 mo.: 92% at 12 mo.: 88%	ND	ND
Takagi et al. [43]	2022	cRP	12	RARP	None	ND	at 24 mo.: >2 pads/day: 1 (8.3)	ND	ND

No.: number, Pts.: patients, MDT: metastasis-directed therapy, RP: radical prostatectomy, PCa: prostate cancer, cRP: cytoreductive radical prostatectomy, IQR: interquartile range, SD: standard deviation, ADT: androgen deprivation therapy, NA: not applicable, ND: no data, ICIQ-SF: International Consultation on Incontinence Questionnaire, IIEF: International Index of Erectile Function.

## Supplementary Materials

The following supporting information can be downloaded at https://www.mdpi.com/article/10.3390/curroncol30020170/s1, Supplementary Figure S1. PRISMA checklist 2009, Supplementary Figure S2. Risk-of-bias assessment of the included RCTs, Supplementary Table S1. Risk-of-bias assessment for NRCTs (ROBINS-I), Supplementary Table S2. Patient characteristics of included population-based studies, Supplementary Table S3. Patient characteristics of included case-control studies.
