# Supplementary material for: An Updated Systematic and Comprehensive Review of Cytoreductive Prostatectomy for Metastatic Prostate Cancer
Source: Curr Oncol. 2023 Feb 10;30(2):2194–216. doi: 10.3390/curroncol30020170 (PMC9955685; doi:10.3390/curroncol30020170)
Supplement: Supplementary file 1 [file curroncol-30-00170-s001.zip › curroncol-2165476-SI.pdf]

Supplementary Information

1. **Supplementary Figure 1.** PRISMA checklist 2009
2. **Supplementary Figure 2.** Risk of bias assessment of the included RCTs
3. **Supplementary Table 1.** Risk of bias assessment for NRCTs (ROBINS-I)
4. **Supplementary Table 2.** Patient characteristics of included population-based studies
5. **Supplementary Table 3.** Patient characteristics of included case-control studies

2. Supplementary Figure 1. PRISMA checklist 2009

Section/topic	#	Checklist item	Reported on page #
**TITLE**			
Title	1	Identify the report as a systematic review, meta-analysis, or both.	1
**ABSTRACT**			
Structured summary	2	Provide a structured summary including, as applicable: background; objectives; data sources; study eligibility criteria, participants, and interventions; study appraisal and synthesis methods; results; limitations; conclusions and implications of key findings; systematic review registration number.	2
**INTRODUCTION**			
Rationale	3	Describe the rationale for the review in the context of what is already known.	5-7
Objectives	4	Provide an explicit statement of questions being addressed with reference to participants, interventions, comparisons, outcomes, and study design (PICOS).	5-7
**METHODS**			
Protocol and registration	5	Indicate if a review protocol exists, if and where it can be accessed (e.g., Web address), and, if available, provide registration information including registration number.	8
Eligibility criteria	6	Specify study characteristics (e.g., PICOS, length of follow-up) and report characteristics (e.g., years considered, language, publication status) used as criteria for eligibility, giving rationale.	8 and 9
Information sources	7	Describe all information sources (e.g., databases with dates of coverage, contact with study authors to identify additional studies) in the search and date last searched.	8 and 9
Search	8	Present full electronic search strategy for at least one database, including any limits used, such that it could be repeated.	8 and 9
Study selection	9	State the process for selecting studies (i.e., screening, eligibility, included in systematic review, and, if applicable, included in the meta-analysis).	8 and 9 Figure.1
Data collection process	10	Describe method of data extraction from reports (e.g., piloted forms, independently, in duplicate) and any processes for obtaining and confirming data from investigators.	9 and 10
Data items	11	List and define all variables for which data were sought (e.g., PICOS, funding sources) and any assumptions and simplifications made.	8 and 9
Risk of bias in individual studies	12	Describe methods used for assessing risk of bias of individual studies (including specification of whether this was done at the study or outcome level), and how this information is to be used in any data synthesis.	10
Summary measures	13	State the principal summary measures (e.g., risk ratio, difference in means).	10
Synthesis of results	14	Describe the methods of handling data and combining results of studies, if done, including measures of consistency (e.g., I^2) for each meta-analysis.	10

Section/topic	#	Checklist item	Reported on page #
Risk of bias across studies	15	Specify any assessment of risk of bias that may affect the cumulative evidence (e.g., publication bias, selective reporting within studies).	10
Additional analyses	16	Describe methods of additional analyses (e.g., sensitivity or subgroup analyses, meta-regression), if done, indicating which were pre-specified.	None
**RESULTS**			
Study selection	17	Give numbers of studies screened, assessed for eligibility, and included in the review, with reasons for exclusions at each stage, ideally with a flow diagram.	12<br>Figure 1
Study characteristics	18	For each study, present characteristics for which data were extracted (e.g., study size, PICOS, follow-up period) and provide the citations.	12<br>Table 1
Risk of bias within studies	19	Present data on risk of bias of each study and, if available, any outcome level assessment (see item 12).	10<br>Supplementary Table 1
Results of individual studies	20	For all outcomes considered (benefits or harms), present, for each study: (a) simple summary data for each intervention group (b) effect estimates and confidence intervals, ideally with a forest plot.	12-14
Synthesis of results	21	Present results of each meta-analysis done, including confidence intervals and measures of consistency.	12-14
Risk of bias across studies	22	Present results of any assessment of risk of bias across studies (see Item 15).	10<br>Supplementary Table 1
Additional analysis	23	Give results of additional analyses, if done (e.g., sensitivity or subgroup analyses, meta-regression [see Item 16]).	None
**DISCUSSION**			
Summary of evidence	24	Summarize the main findings including the strength of evidence for each main outcome; consider their relevance to key groups (e.g., healthcare providers, users, and policy makers).	16-19
Limitations	25	Discuss limitations at study and outcome level (e.g., risk of bias), and at review-level (e.g., incomplete retrieval of identified research, reporting bias).	18 and 19
Conclusions	26	Provide a general interpretation of the results in the context of other evidence, and implications for future research.	19 and 20
**FUNDING**			
Funding	27	Describe sources of funding for the systematic review and other support (e.g., supply of data); role of funders for the systematic review.	20,21

2. Supplementary Figure 2. Risk of bias assessment of the included RCTs; (A) Risk of bias summary, (B) Risk of bias graph

(A)

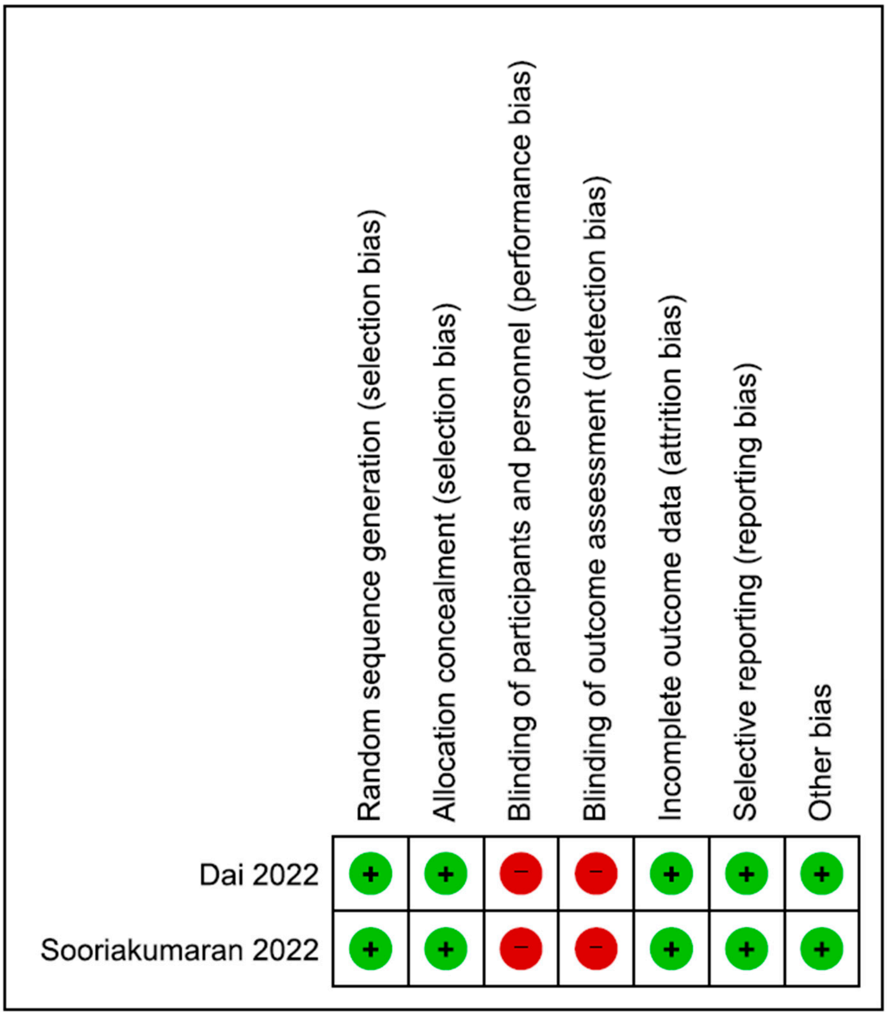

(B)

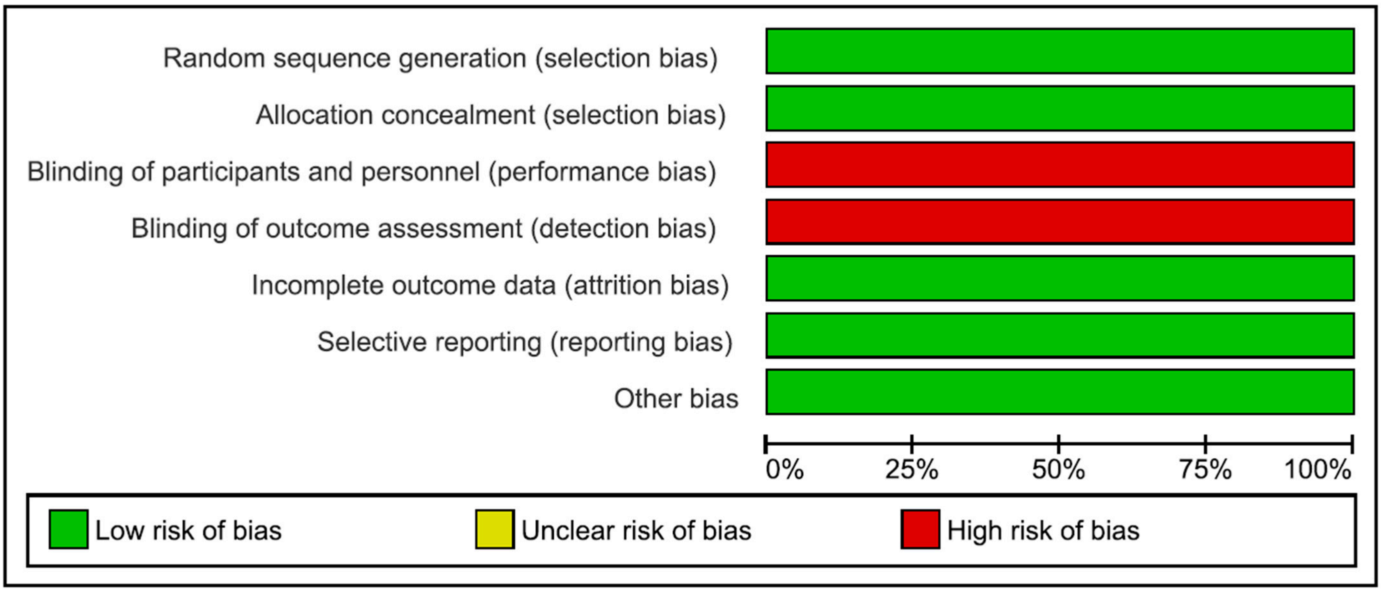

3. Supplementary Table 1. Risk of bias assessment for NRCTs (ROBINS-I)

**Supplementary Table.1 Risk of bias assessment for NRCTs (ROBINS-I)**									
**Authors**	Year	Confounding	Participants' selection	Classification of interventions	Deviations from intended intervention	Missing data	Measurement of outcomes	Selection of the reported result	Overall
Population-based study									
**Culp**	2014	Moderate	Moderate	Low	Low	Low	Moderate	Low	Moderate
**Gratzke**	2014	Moderate	Moderate	Low	Low	Low	Moderate	Low	Moderate
**Antwi**	2014	Moderate	Moderate	Low	Low	Low	Moderate	Low	Moderate
**Satkunasivam**	2015	Moderate	Moderate	Low	Low	Low	Moderate	Low	Moderate
**Parikh**	2017	Moderate	Moderate	Low	Low	Low	Moderate	Low	Moderate
**Jin S**	2020	Moderate	Moderate	Low	Low	Low	Moderate	Low	Moderate
**Jin K**	2020	Moderate	Moderate	Low	Low	Low	Moderate	Low	Moderate
**Guo**	2021	Moderate	Moderate	Low	Low	Low	Moderate	Low	Moderate
**Stolzenbach**	2021	Moderate	Moderate	Low	Low	Low	Moderate	Low	Moderate
Case-control or single arm study									
**Heidenreich**	2015	Moderate	Moderate	Moderate	Low	Moderate	Low	Low	Moderate
**Gandaglia**	2017	Moderate	Moderate	Moderate	Low	Moderate	Low	Low	Moderate
**Poelaert**	2017	Moderate	Moderate	Moderate	Low	Moderate	Moderate	Low	Moderate
**Moschini**	2017	Moderate	Moderate	Moderate	Low	Moderate	Moderate	Low	Moderate
**Steuber**	2017	Moderate	Moderate	Moderate	Low	Moderate	Moderate	Moderate	Moderate
**Buelens**	2022	Moderate	Moderate	Moderate	Low	Moderate	Moderate	Low	Moderate
**Mistretta**	2022	Moderate	Moderate	Moderate	Low	Moderate	Moderate	Low	Moderate
**Knipper**	2020	Moderate	Moderate	Moderate	Low	Moderate	Low	Low	Moderate
**Lumen**	2021	Moderate	Moderate	Moderate	Low	Moderate	Moderate	Low	Moderate
**Chaloupka**	2021	Moderate	Moderate	Moderate	Low	Moderate	Low	Low	Moderate

**Heidenreich**	2018	Moderate	Low	Low	Low	Moderate	Low	Low	Moderate
**Xue**	2020	Moderate	Low	Low	Low	Moderate	Moderate	Low	Moderate
**Mandel**	2021	Moderate	Low	Low	Low	Moderate	Moderate	Low	Moderate
**Babst**	2021	Moderate	Low	Low	Low	Moderate	Low	Moderate	Moderate
**Kim**	2022	Moderate	Moderate	Low	Low	Moderate	Moderate	Low	Moderate
**Takagi**	2022	Moderate	Moderate	Low	Low	Moderate	Moderate	Low	Moderate
**NRCTs: non-randomized comparative studies, ROBINS-I: Risk Of Bias In Non-Randomized Studies -of Interventions**									

4. **Supplementary Table 2.** Patient characteristics of included population-based studies

**Supplementary Table 2. Patient characteristics of included population-based studies**														
**Author**	Year	Comparisons	No. of patients	Recruitment year	Age at local treatment, years	Performance status or comorbidity	cTstage, n (%)	cNstage, n (%)	Biopsy Gleason score*, n (%)	PSA (ng/ml) or n (%)	Metastatic site, n (%)	Detailed metastatic site, n (%)	ADT, n (%)	EBRT received, n (%)
**Culp (SEER)**	2014	NLT	7811	2004-2010	Median (IQR): 72 (63-80)	ND	T1/T2: 4318 (55) T3/T4: 1562 (20) Unknown: 1931(25)	N0: 3807 (49) N1: 1514 (19) Unknown: 2490 (32)	Well to moderate: 439 (5.6) Poor: 5673 (73) Unknown: 1699 (22)	<10/10-19/20-29/>30: 632(8.1)/871(11)/558(7.1)/4815(62)	M1a: 463 (5.9) M1b: 5469 (70) M1c: 1879 (24)	ND	ND	0
		cRP	245		Median (IQR): 62 (58-67)		T1/T2: 130 (53) T3/T4: 113 (46) Unknown: 2 (0.8)	N0: 165 (67) N1: 68 (28) Unknown: 12 (32)	Well to moderate: 51 (21) Poor: 187 (76) Unknown: 7 (2.9)	<10/10-19/20-29/>30: 115(47)/50(20)/17(6.9)/32(13)	M1a: 24 (9.8) M1b: 150 (61) M1c: 71 (29)			41 (17)
		BT	129		Median (IQR): 68 (61-74)		T1/T2: 91 (71) T3/T4: 20 (16) Unknown: 18 (14)	N0: 3807 (49) N1: 1514 (19) Unknown:	Well to moderate: 24 (19) Poor: 94 (73) Unkno	<10/10-19/20-29/>30: 45(35)/19(15)/11(8.5)/44 (34)	M1a: 16 (12) M1b: 75 (58) M1c: 38 (30)			54 (42)

								2490 (32)	wn: 11 (8.5)					
**Gratzke (Munich Cancer Registry)**	2014	NLT	1075	1998-2010	ND	ND	ND	ND	ND	ND	ND	ND	ND	ND
		cRP	74											
		RT	389											
**Antwi (SEER)**	2014	NLT	7516	2004-2010	<65: 2305 (31)<br>>65: 5211 (69)	ND	ND	ND	Well to moderate: 391 (5.2)<br>Poor: 5433 (72)<br>Unknown: 1692 (23)	<10: 89 (1.2)<br>>10: 6805 (91)	M1a: 433 (5.8)<br>M1b: 5264 (70)<br>M1c: 1819 (24)	ND	ND	0
		cRP	222		<65: 142 (64)<br>>65: 80 (36)				Well to moderate: 39 (17)<br>Poor: 175 (79)<br>Unknown: 8 (3.6)	<10: 10 (4.5)<br>>10: 195 (88)	M1a: 20 (9.0)<br>M1b: 139 (63)<br>M1c: 63 (28)			34 (15)
		BT	120		<65: 40 (33)<br>>65: 80 (67)				Well to moderate: 22 (18)<br>Poor: 85 (71)<br>Unkno	<10: 4 (3.3)<br>>10: 109 (91)	M1a: 18 (15)<br>M1b: 68 (57)<br>M1c: 34 (28)			51 (42)

									wn: 13 (11)					
**Satkunasivam (SEER)**	2015	NLT	3827	2004-2009	Mean± SD: 78.2±7.2	CCI 0/1/2/3<: 2462(64)/757(20)/331(9)/277(7)	T1/T2: 839(22)/1282(33) T3/T4: 298 (8)/461 (12) Unknown: 947 (25)	N0: 1930 (50) N1: 577 (15) Unknown: 1320 (34)	<6: 167 (4) 7: 569 (15) 8-10: 2042 (53)	Mean±SD: 590±380	M1a: 190 (5) M1b: 2570 (67) M1c: 922 (24)	ND	None: 1132 (30) Orchiectomy: 331 (9) GnRH agonist: 2330 (61)	ND
		cRP	47		Mean± SD: 73.0±6.0	CCI 0/1/2/3<: 32(68)/9(19)/4(9)/2(4)	T1/T2: 0/21(45) T3/T4: 19 (40)/6 (13) Unknown: 1(2)	N0: 34 (72) N1: 10 (21) Unknown: 3 (6)	<6: 5 (11) 7: 22 (47) 8-10: 19 (40)	Mean±SD: 181±263	M1a: 3(6) M1b: 26 (55) M1c: 17 (36)		None: 27 (57) Orchiectomy: 3 (6) GnRH agonist: 16 (34)	
		IMRT	88		Mean± SD: 74.2±6.1	CCI 0/1/2/3<: 60(68)/19(22)/4(5)/5(6)	T1/T2: 27(31)/36(41) T3/T4: 10 (11)/9 (10) Unknown: 6(7)	N0: 59 (67) N1: 11 (13) Unknown: 18 (20)	<6: 10 (11) 7: 24 (27) 8-10: 43 (49)	Mean±SD: 282±338	M1a: 4 (5) M1b: 65 (74) M1c: 16 (18)		None: 30 (34) Orchiectomy: 0 GnRH agonist: 56 (64)	
		CRT	107		Mean± SD: 76.4±6.3	CCI 0/1/2/3<: 67(63)/25(23)/10(9)/5 (5)	T1/T2: 31(29)/28(26) T3/T4: 9 (8)/17 (16) Unknown: 22(21)	N0: 63 (59) N1: 15 (14) Unknown: 29 (27)	<6: 8 (7) 7: 22 (21) 8-10: 59 (55)	Mean±SD: 531±369	M1a: 4 (4) M1b: 72 (67) M1c: 31 (29)		None: 13 (12) Orchiectomy: 5 (5) GnRH agonist: 88 (82)	
**Parikh (NCDB)**	2017	NLT	5224	2004-2013	Median: 72	CCI 0/1-2/3<: 3803(73)/1001(19)/420(8.0)	T1/T2: 1404(27)/855 (16) T3/T4: 560(11)/1039	N0: 2164 (42) N1: 1087	<6: 33 (0.6) 7: 214 (4.0) 8-10:	<10: 81 (4.1) 11-20: 72 (4.0) >20: 1834 (92)	M1a: 12 (0.2) M1b: 2286	ND	None: 9 (5.1) Yes: 169 (95)	ND

							(20)<br>Unknown: 1366 (26)	(21)<br>Unknown: 1889 (37)	4969 (95)		(44)<br>M1c: 334 (6.7)			
		cRP	622			CCI 0/1-2/3<: 492(79)/107(17)/23(3.7)	T1/T2: 195(31)/135(22)<br>T3/T4: 62(10)/46(7.4)<br>Unknown: 184 (30)	N0: 323 (53)<br>N1: 62 (10)<br>Unknown: 228 (37)	<6: 19 (3.1)<br>7: 67 (11)<br>8-10: 536 (86)	<10: 17 (4.2)<br>11-20: 16 (3.9)<br>>20: 373 (92)	M1a: 1(0.2)<br>M1b: 168 (28)<br>M1c: 15 (2.5)		None: 6 (16)<br>Yes: 32 (84)	
		CRT	153			CCI 0/1-2/3<: 125(82)/18(12)/10(6.5)	T1/T2: 40(26)/21(14)<br>T3/T4: 21(14)/48(31)<br>Unknown: 23 (15)	N0: 70 (46)<br>N1: 41 (27)<br>Unknown: 40 (26)	<6: 1 (0.7)<br>7: 4 (2.6)<br>8-10: 148 (97)	<10: 2 (2.5)<br>11-20: 5 (6.2)<br>>20: 74 (91)	M1a: 0<br>M1b: 55 (36)<br>M1c: 10 (6.6)		None: 6 (19)<br>Yes: 25 (81)	
		IMRT	52			CCI 0/1-2/3<: 41(79)/6(12)/5(9.6)	T1/T2: 11(21)/12(23)<br>T3/T4: 8(15)/20(38)<br>Unknown: 1 (1.9)	N0: 33 (63)<br>N1: 8 (15)<br>Unknown: 11 (21)	<6: 0<br>7: 2 (3.9)<br>8-10: 50 (96)	<10: 2 (5.7)<br>11-20: 2 (5.7)<br>>20: 31 (89)	M1a: 0<br>M1b: 28 (54)<br>M1c: 2 (3.9)		None: 3 (30)<br>Yes: 7 (70)	
**Jin S (SEER)**	2020	NLT	5628	2010-2014	Median (IQR): 70 (62-78)	ND	T1/T2: 1469(26)/1757(31)<br>T3/T4: 616(11)/610(11)<br>Unknown: 66(1.2)	N0: 3035 (54)<br>N1: 1452 (26)<br>Unknown: 1141 (20)	<6: 124 (2.2)<br>7: 674 (12)<br>8-10: 3567 (63)	<10/10-19/20-29/>30: 429(7.6)/451(8.0)/348(6.2)/3813(68)	M1a: 371 (6.6)<br>M1b: 4078 (73)<br>M1c: 963 (17)	Bone: 4888 (87)<br>Liver: 237 (4.2)<br>Lung: 421 (7.5)	ND	ND
		cRP	159		Median (IQR): 62 (57-67)		T1/T2: 7(4.4)/51(32)<br>T3/T4: 88(55)/9(5.7)	N0: 99 (62)<br>N1: 55 (35)<br>Unkno	<6: 13 (8.2)<br>7: 54 (34)	<10/10-19/20-29/>30: 86(54)/30(19)/12(7.5)/20 (13)	M1a: 20 (13)<br>M1b: 105	Bone: 114 (72)<br>Liver: 6 (3.8)		

							Unknown: 4(2.5)	wn: 5 (3.1)	8-10: 76 (48)		(66) M1c: 28 (18)	Lung: 10 (6.3)		
		BT	62		Median (IQR): 65 (56-70)		T1/T2: 30(48)/14(23) T3/T4: 3(4.8)/4(6.5) Unknown: 11(18)	N0: 44 (71) N1: 8 (13) Unknown: 10 (16)	<6: 8 (13) 7: 14 (23) 8-10: 32 (52)	<10/10-19/20-29/>30: 21(34)/7(11)/4(6.5)/24(39)	M1a: 4 (6.5) M1b: 44 (71) M1c: 11 (18)	Bone: 48 (77) Liver: 2 (3.2) Lung: 1 (1.6)		
**Jin K (SEER)**	2020	NLT	18857	2004-2015	Mean±SD: 71.5±11.0	ND	T1/T2: 4760(25)/5839(31) T3/T4: 1679(8.9)/2326(12) Unknown: 4253(23)	N0: 9682 (51) N1: 4307 (23) Unknown: 4868 (26)	<6: 845 (4.5) 7: 1421 (7.5) 8-10: 4578 (24)	Mean±SD: 67.8±36.6	M1a: 1207 (6.4) M1b: 13704 (73) M1c: 3946 (21)	ND	ND	NA
		cRP	435		Mean±SD: 62.8±8.0		T1/T2: 11(2.5)/153(35) T3/T4: 199(46)/64(15) Unknown: 8(1.8)	N0: 252 (58) N1: 162 (37) Unknown: 21 (4.8)	<6: 27 (6.2) 7: 58 (13) 8-10: 76 (17)	Mean±SD: 19.4±23.7	M1a: 58 (13) M1b: 309 (71) M1c: 68 (16)			
		RT	320		Mean±SD: 67.7±10.0		T1/T2: 119(37)/87(27) T3/T4: 34(11)/26(8.1) Unknown: 54(17)	N0: 210 (66) N1: 41 (13) Unknown: 69 (22)	<6: 30 (9.4) 7: 42 (13) 8-10: 83 (26)	Mean±SD: 42.1±38.7	M1a: 30 (9.4) M1b: 217 (68) M1c: 73 (23)			

**Guo (SEER)**	2021	cRP	481 (148) **	2004-2016	Median (IQR): 65 (58-69)	ND	T1/T2: 8(5.4)/86(58) T3/T4: 48(32)/6(4.1)	ND	<6: 15 (10) 7: 40 (27) 8-10: 93 (63)	Median (IQR): 16.6 (6.3-31.0)	M1a: 26 (18) M1b: 94 (64) M1c: 28 (19)	ND	ND	ND
		RT	203 (148) **		Median (IQR): 65 (56-72)		T1/T2: 18(12)/72(49) T3/T4: 48(32)/10(6.8)		<6: 18 (12) 7: 40 (27) 8-10: 90 (61)	Median (IQR): 15.0 (5.8-38.7)	M1a: 24 (16) M1b: 94 (64) M1c: 30 (20)			
**Stolzenbach (SEER)**	2021	cRP	954	2004-2016	Median (IQR): 65 (59-72)	ND	T1/T2: 534 (56) T3/T4: 199 (21) Unknown: 221 (23)	N0/NX: 620 (65) N1: 334 (35)	I / II / III/IV/ V: 39 (4.1)/ 94(10)/ 85(8.9)/ 152(16)/ 339 (36) Unknown: 245 (26)	Median (IQR): 32.4 (9.2-98)	M1a: 114 (12) M1b: 840 (88)	ND	ND	ND
		RT	3326		Median (IQR): 67 (60-76)		T1/T2: 1917 (58) T3/T4: 768 (23) Unknown: 641 (19)	N0/NX: 2479 (74.5) N1: 847(25.5)	I / II / III/IV/ V: 99 (3.0)/ 213(6.4)/	Median (IQR): 97.9 (23-98)	M1a: 182 (5.5) M1b: 3144 (94.5)			

									272(8.2)/ 637(19)/ 1353 (41) Unknown: 752 (23)					

No.; Number, Pts.: Patients, NCDB; National Cancer Database, SEER; Surveillance, Epidemiology and End Results, LT; Local Therapy, NLT: No Local Therapy, RT; Radiotherapy, EBRT; External Beam Radiotherapy, BT; Brachytherapy, IMRT; Intensity-modulated Radiotherapy, CRT; Conformal Radiation Therapy, cRP; Cytoreductive Radical Prostatectomy, IQR; Interquartile range, SD; Standard Deviation, ADT; Androgen Deprivation Therapy, GnRH; Gonadotropin Releasing Hormone, NA; Not Applicable, ND; No Data, PSA; Prostate Specific Antigen, CCI; Charlson Comorbidity Index,

*Before 2014, studies from SEER database described as differentiation.
**Described as number of PSM cohorts

5. Supplementary Table 3. Patient characteristics of included case-control or single arm studies

Supplementary Table 3. Patient characteristics of case-control or single arm studies

**Author**	Year	Comparisons	No. of patients	Age, years	Performance status or comorbidity	cTstage, n (%)	Biopsy Gleason score (Gleason group), n (%)	PSA, ng/ml	Metastatic burden	Metastatic site, n (%)	Neoadjuvant HT, n (%)
<u>Comparative studies between cRP vs. No cRP</u>											
**Heidenreich**	2015	cRP	23	Mean(range):<br>61 (42-69)	CCI (mean, range): 6.6 (6-9)	cT2: 7 (30)<br>cT3a/b: 16 (70)<br>cT4: 0	6/7/8/9/10:<br>0/5(22)/7(30)/7(30)/4(17)	Mean(range):<br>135.2 (3.5-150.4)	Mean bone metastases (range): 2.1 (1-3)	LN:<br><2cm: 3 (13)<br>2-3cm: 1 (4.3)	ND
		No cRP	38	Mean(range):<br>63.9 (47-83)	CCI (mean, range): 7.1 (6-11)	cT2: 10 (26)<br>cT3a/b: 24 (63)<br>cT4: 4 (11)	6/7/8/9/10:<br>4 (11)/11(29)/11(29)/8(21)/4(11)	Mean(range):<br>105.9 (45-195)	Mean bone metastases (range): 2.5 (1-5)	LN:<br><2cm: 6 (16)<br>2-3cm: 2 (5.3)	
**Poelaert (LoMP trial)**	2017	cRP	17	Mean±SD:<br>**64±8**	ND	cT1-2: 8 (47)<br>cT3a/b: 2(12)/7(41)<br>cT4: 0	1/2/3/4/5:<br>0/2(12)/2(12)/5(29)/8(47)	**Mean(range):<br>16 (4.6-75)**	**Low-volume: 16 (94)**	M1a: 9 (53)<br>M1b: 8 (47)<br>M1c: 0	ND
		No cRP	29	Mean±SD:<br>72±10		cT1-2: 3 (10)<br>cT3a/b: 10(35)/3(10)<br>cT4: 13 (45)	1/2/3/4/5:<br>1(3.4)/0/1(3.4)/8(28)/19(66)	Mean(range):<br>156 (5.2-3092)	Low-volume: 9 (31)	M1a: 4 (14)<br>M1b: 21 (72)<br>M1c: 4 (14)	
**Moschini**	2017	cRP	31	Median (IQR):<br>62 (56-66)	ND	<cT2: 13 (42)<br>cT3-T4:18 (58)	3+5/4+3/4+4/4+5/5+5<br>2(6.5)/0/10 (32)/15 (48)/4(13)	Median (IQR):<br>24 (12.4-107)	ND	M1a: 17 (55)<br>M1b: 14 (45)	ND
		No cRP	16	Median (IQR):<br>59 (54-59)		<cT2: 12 (75)<br>cT3-T4: 2 (13)	3+5/4+3/4+4/4+5/5+5<br>0/2(13)/5 (31)/8 (50)/1(6.3)	Median (IQR):<br>76.5 (2.7-218)		M1a: 8 (50)<br>M1b: 8 (50)	

**Steuber**	2017	cRP	43	Median: 65	ND	<cT2: 46.5%<br>cT3a/b: 53.5%	6/7/8/9/10:<br>0/30%/30%/35%/4.7%	Median:29	No. of bone metastasis 1/2/3: 67%/21%/12%	NA	44%
		No cRP	40	Median: 70		<cT2: 22.5%<br>cT3a/b: 77.5%	6/7/8/9/10:<br>2.5%/12.5%/32.5%/40%/12.5%	Median:42.5	No. of bone metastasis 1/2/3: 41%/31%/28%		NA
**Buelens (LoMP trial)**	2022	cRP	40	Median (IQR):<br>**66 (59-73)**	**ECOG 0-1: 39 (98)<br>ECOG 2-4: 1 (2.5)**	cT1-2: 12 (30)<br>cT3-4: 28 (70)	1-3/ 6(15)<br>4-5/ 34 (85)	**Median (IQR):<br>19.6 (11.2-45.2)**	**Low-volume: 32 (80)<br>Median number of bone metastasis (IQR): 2 (1-6)**	M1a: 16 (40)<br>M1b: 22 (55)<br>M1c: 2 (5)	17 (43)
		No cRP	40	Median (IQR):<br>76 (69-83)	ECOG 0-1: 33 (83)<br>ECOG 2-3: 7 (18)	cT1-2: 7 (18)<br>cT3-4: 33 (83)	1-3/ 5(13)<br>4-5/ 35 (83)	Median (IQR):<br>166 (37.1-592.7)	Low-volume: 14 (35)<br>Number of bone metastasis (median, IQR): 8 (4-18)	M1a: 6 (14)<br>M1b: 23 (58)<br>M1c: 11 (28)	33 (83)
**Mistretta**	2022	cRP	40	Median (IQR):<br>67 (58-68)	CCI<br><2: 26 (65)<br>>3: 14 (35)	cT1-2: 20 (50)<br>cT3-4: 20 (50)	1-3: 18 (45)<br>4-5: 22 (55)	**Median (IQR):<br>14 (9-29)**	ND	M1a: 16 (40)<br>M1b: 24 (60)	ND
		No cRP	34	Median (IQR):<br>64 (60-74)	CCI<br><2: 16 (47)<br>>3: 18 (53)	cT1-2: 12 (35)<br>cT3-4: 22 (65)	1-3: 9 (26)<br>4-5: 23 (68)	Median (IQR):<br>87 (35-186)		M1a: 7 (21)<br>M1b: 27 (79)	
<u>**RCT(Phase1/2)** ***for cRP vs. No cRP***</u>											

**Sooriakumaran (TRoMbone)**	2022	cRP	25	Median (IQR):<br>65.4 (62.5-69.3)	No comorbidities:<br>21 (84)	cT2c: 3 (12)<br>cT3: 22 (88)	3+4: 1 (4)<br>4+3: 6 (24)<br>8: 7 (28)<br>9-10: 11 (44)	Median (IQR):<br>14 (2.7-40)	Number of bone metastasis (median,IQR):<br>1.0 (1.0-2.0)	N0: 16 (64)<br>N1: 9 (36)	All
		No cRP	25	Median (IQR):<br>66 (60.2-71.2)	No comorbidities:<br>23 (92)	cT2c: 2 (8)<br>cT3: 23 (92)	3+4: 2 (8)<br>4+3: 5 (20)<br>8: 6 (24)<br>9-10: 12 (48)	Median (IQR):<br>16.5 (8.2-37.5)	Number of bone metastasis (median,IQR):<br>1.0 (1.0-2.0)	N0: 10 (40)<br>N1: 15 (60)	
<u>Comparative studies between cRP vs. RT</u>											
**Knipper**	2020	cRP	78	Median (IQR):<br>64 (59-69)	WHO PS 0/1-2:<br>78(100)/0	cT1/T2:27(35)/31(40)<br>cT3/T4:16 (20)/0	<7: 18 (23)<br>8-10: 60 (77)	Median (IQR):<br>35 (13-55)	All low-volume	Bone: 78 (100)<br>Distant LN:0	ND
		STAMPEDE arm H (low volume with RT)	410	Median (IQR):<br>68 (63-73)	WHO PS 0/1-2:<br>313(76)/97(24)	cT1/T2:6(2)/32(8)<br>cT3/T4:261(66)/94 (24)	<7: 84 (21)<br>8-10: 308 (79)	Median (IQR):<br>55 (23-138)		Bone: 311 (76)<br>Distant LN:149 (36)	
<u>RCT (Phase2) assessing LT (including 85% of cRP) vs. NLT</u>											
**Dai**	2022	LT	100 (85)*	Median (IQR):<br>67 (62-71)	ND	<T2c: 14<br>T3a-b: 72<br>T4: 14	<7: 14<br>8-10: 86	Median (IQR):<br>90 (35-236)	ND	N1: 37<br>Distant LN: 10<br>Bone: 97	63 (74)
		NLT	100	Median (IQR):<br>69 (64-73)		<T2c: 20<br>T3a-b: 61<br>T4: 19	<7: 12<br>8-10: 85	Median (IQR):<br>102 (49-254)		N1: 42<br>Distant LN: 20<br>Bone: 95	NA
<u>Comparative studies between cRP vs. RT vs. NLT</u>											
**Lumen (LoMP trial)**	2021	cRP	48	Median (IQR):<br>64 (59-72)	ECOG 0-1: 47 (98)<br>ECOG 2-3: 1 (2.1)	cT1-2: 17 (35)<br>cT3-4: 31 (65)	1/2/3/4/5:<br>1(2.1)/3(6.3)/6(13)/10(21)/28 (58)	Median (IQR):<br>19 (11-42)	All low-volume	M1a: 23 (48)<br>M1b: 25 (52)	40 (83)

		RT	26	Median (IQR):<br>70 (61-76)	ECOG 0-1: 25 (96)<br>ECOG 2-3: 1 (3.8)	cT1-2: 5 (19)<br>cT3-4: 21 (81)	1/2/3/4/5:<br>0/1(3.8)/2(7.7)/5(19)/18(69)	Median (IQR):<br>40 (15-67)		M1a: 9 (35)<br>M1b: 17 (65)	26 (100)
		NLT	35	Median (IQR):<br>74 (69-84)	ECOG 0-1: 29 (83)<br>ECOG 2-3: 6 (17)	cT1-2: 4 (11)<br>cT3-4: 31 (89)	1/2/3/4/5:<br>3(9.4)/2(6.3)/3(9.4)/11(34)/13 (41)	Median (IQR):<br>47 (17-156)		M1a: 10 (29)<br>M1b: 25 (71)	30 (86)
<u>Comparative studies between cRP vs. RP for localized PCa</u>											
**Chaloupka**	2021	cRP	79	Median (IQR):<br>66 (60-72)	ASA score>2: 61%	ND	ND	Median (IQR):<br>21.1 (13-55)	NA	NA	None
		RP	332	Median (IQR):<br>68 (60-73)	ASA score>2: 45%			Median (IQR):<br>35 (13-55)			
<u>Single arm or only including cRP cohort</u>											
**Heidenreich**	2018	cRP	113	Median(range):<br>60.2 (42-69)	ND	ND	6/7/8/9/10:<br>4(3.5)/18(16)/42(37)/40(35)/9 (7.9)	Median (range):<br>8.0 (2.5-390)	Low-volume: 88 (78)<br>High-volume: 25 (22)	ND	ADT alone: 69 (61)<br>ADT+DOC: 11 (9.7)
**Xue**	2020	cRP + MDT	26	Median(range):<br>65.5 (54-78)	ND	cT1-2: 5 (19)<br>cT3a: 13 (50)<br>cT3b-4: 8 (31)	1/2/3/4/5:<br>7(27)/6(23)/5(19)/5(19)/3(12)	Median (range):<br>35.3 (8.9-213.5)	Number of bone metastasis<br>1-3: 20 (77)<br>4-5: 6 (23)	cN0: 20 (77)<br>cN1: 6 (23)	5 (8.6%)
		cRP only	32	Median(range):<br>67.5 (51-79)		cT1-2: 5 (16)<br>cT3a: 12 (38)<br>cT3b-4: 15 (47)	1/2/3/4/5:<br>10(31)/7(22)/5(16)/6(19)/4(13)	Median (range):<br>36.4 (9.7-756.3)	Number of bone metastasis<br>1-3: 22 (69)<br>4-5: 10 (31)	cN0: 21 (66)<br>cN1: 11 (34)	
**Mandel (ProMPT trial)**	2021	cRP (assessing CTC as prognostic value)	33	ND	ND	<cT3a: 16 (49)<br>>cT3a: 17 (52)	<5: 20 (61)<br>5: 13 (39)	Median (IQR):<br>29.7 (10.6-54.3)	Number of bone metastasis<br>1: 20 (61)<br>2: 9 (27)<br>3: 4 (12)	ND	ND

**Babst**	2021	cRP (assessing the impact of upfront DOC-based doublet therapy)	38	Median (IQR): 57 (54-64)	ASA score Median (IQR): 2 (2-2)	ND	6/7/8/9/10: 1(2.6)/4(11)/9(24)/21(55)/3(7.9)	Median (IQR): 65 (35-125) at diagnosis 1.0 (0.3-1.7) at preoperative	Low-volume: 28 (74) High-volume: 10 (26)	cN1: 33 (87) M1b: 23 (61) M1c: 2(5)	All
**Kim**	2022	cRP	32	Median (IQR): 64.5 (57.5-70)	**ND**	ND	1/2/3/4/5: 1(3)/6(19)/4(12)/7(22)/12(38)	Median (IQR): 22.9 (11.1-103.9)	ND	N1M0: 7 (22) N0M1: 10 (31) N1M1: 15 (47)	ND
**Takagi**	2022	cRP (assessing the feasibility of RARP)	12	Median (IQR): 74.5 (62-79)	ND	T2: 5 (42) T3: 5 (42) T4: 2 (17)	4+3: 2 (17) 4+4: 2 (17) 4+5: 6 (50) 5+4: 2 (17)	Median (IQR): 185.3 (34.9-333.4)	Number of bone metastasis 1: 5 (42) 3: 5 (42) >5: 2 (17)	cN1:7(58) M1b: 12(100)	All Duration (mo, median, IQR): 7.3 (6.1-27)

No.; Number, Pts.: Patients, LT; Local Therapy, NLT: No Local Therapy, RT; Radiotherapy, EBRT; External Beam Radiotherapy, BT; Brachytherapy, MDT; metastasis-directed therapy, PSM; Propensity Score Matching, ECOG-PS; Eastern Cooperative Oncology Group Performance Status, ASA; American Society of Anaesthesiology, cRP; Cytoreductive Radical Prostatectomy, SOC; Standard Of Care, RARP; Robot-assisted Radical Prostatectomy, IQR; Interquartile range, SD; Standard Deviation, ADT; Androgen Deprivation Therapy, DOC; Docetaxel, NA; Not Applicable, ND; No Data, PSA; Prostate Specific Antigen, CTC; Circulating Tumor Cell

*85 patients underwent cRP
